# High-Throughput Screening of Free Proline Content in Rice Leaf under Cadmium Stress Using Hyperspectral Imaging with Chemometrics

**DOI:** 10.3390/s20113229

**Published:** 2020-06-05

**Authors:** Tingting Shen, Chu Zhang, Fei Liu, Wei Wang, Yi Lu, Rongqin Chen, Yong He

**Affiliations:** 1 College of Biosystems Engineering and Food Science, Zhejiang University, 866 Yuhangtang Road, Hangzhou 310058, China; ttingshen@zju.edu.cn (T.S.); chuzh@zju.edu.cn (C.Z.); wwang_2017@zju.edu.cn (W.W.); yilu@zju.edu.cn (Y.L.); chenrq@zju.edu.cn (R.C.); yhe@zju.edu.cn (Y.H.); 2 Key Laboratory of Spectroscopy Sensing, Ministry of Agriculture and Rural Affairs, Hangzhou 310058, China; 3 Huanan Industrial Technology Research Institute of Zhejiang University, Guangzhou 510700, China

**Keywords:** hyperspectral image, free proline, rice leaf, cadmium stress, chemometrics, phenotype

## Abstract

Tracking of free proline (FP)—an indicative substance of heavy metal stress in rice leaf—is conducive to improve plant phenotype detection, which has important guiding significance for precise management of rice production. Hyperspectral imaging was used for high-throughput screening FP in rice leaves under cadmium (Cd) stress with five concentrations and four periods. The average spectral of rice leaves were used to show differences in optical properties. Partial least squares (PLS), least-squares support vector machine (LS-SVM) and extreme learning machine (ELM) models based on full spectra and effective wavelengths were established to detect FP content. Genetic algorithm (GA), competitive adaptive weighted sampling (CARS) and PLS weighting regression coefficient (Bw) were compared to screen the most effective wavelengths. Distribution map of the FP content in rice leaves were obtained to display the changes in the FP of leaves visually. The results illustrated that spectral differences increased with Cd stress time and FP content increased with Cd stress concentration. The best result for FP detection is the ELM model based on 27 wavelengths selected by CARS and *R_p_* is 0.9426. Undoubtedly, hyperspectral imaging combined with chemometrics was a rapid, cost effective and non-destructive technique to excavate changes of FP in rice leaves under Cd stress.

## 1. Introduction

Free proline (FP) is a dipolar nitrogen-containing compound widely present in plant cells. It has high water solubility and is an organic osmotic protective agent. It can protect the cell membrane system, maintain the structure of intracellular enzymes, reduce the degradation of intracellular proteins and remove aerobic free radicals [1,2,3]. As significant environmental pollutants, the toxicity of heavy metals are stresses to plant growth, especially crop quality and safety [4]. Heavy metal stress interferes with water absorption and ion channels in rice plants, so water deficits and large accumulations of free proline often occur in plants [5]. Choudhary et al. [4] found that the higher the concentration of heavy metal stress, the more FP in the plant. There is a correlation between free proline and free radical content, which can be used as an aerobic free radical scavenger; it also pointed out that proline is an amino acid. It has a certain chelating ability and may combine with metal ions to form a plant survival defense mechanism, but this conjecture has not been verified. Zouari et al. [6] pointed out that the addition of foreign aid proline would slow down the oxidative damage to plants caused by cadmium (Cd) stress, and the antioxidant enzyme activity, photosynthetic activity and plant growth in the leaves were alleviated, so it was inferred that the free proline content can be targeted to the heavy metal stress response.

Rice is a staple food for a large part of the human population in the world, especially in Asia. Cd pollution is the most influential in the reported heavy metal pollution incidents in rice [6]. With the development of modern sensors, researchers can capture thousands of phenotypes of a plant directly or indirectly [7]. Using these phenotypes, researchers can evaluate the plant growth performances and make decisions, including analyzing the plants under stresses [8]. Undoubtedly, FP is an important phenotype of rice leaves under heavy metal stress and monitoring the change of FP content in rice leaves under heavy metal stress is significant for providing guides for rice growth management.

The traditional method for detecting the content of physiological indicators is demanding, requiring a low-temperature environment and strict operation procedures, because FP in fresh leaves is easily decomposed. Under acidic conditions, ninhydrin reacts with proline to form a red compound. Traditional detection mainly uses this principle to detect the content of free proline by a spectrophotometer or microplate reader. Although results of the traditional method are accurate, the detection process takes time and effort. Real-time monitoring and high-throughput detection are not easy, and it is impossible to observe differences in the distribution of adverse physiological indicators on the surface of rice leaves.

Hyperspectral imaging is a rapid, nondestructive and high-throughput phenotyping technique, which has been widely used to study plant phenotypes [9,10,11]. The most noticed advantage is ‘map and spectrum integration’. Spectral reflected the internal physiological, biochemical and structural characteristics of rice leaf tissue while acquiring the image of the leaf appearance contained color, texture, etc. Phenotypes as optical properties and some spatial features can be directly derived from the hyperspectral images, and much more phenotype features can be further obtained indirectly by analyzing the images and spectra [12]. Kong et al. [13] used the hyperspectral imaging system in the 400–1000 nm to quickly detect the content of malondialdehyde, selected 23 optimal characteristic wavelengths, to establish a fast prediction model with correlation of the prediction set 0.929 and established the distribution map of malondialdehyde on rape leaves. Zhang et al. [14] used hyperspectral imaging systems combined with chemometrics methods to quickly estimate the soluble sugar content of four different growth extremes of rapeseed leaves, and tried to predict the visual distribution of soluble sugars in rapeseed leaves. The above research shows that the hyperspectral technology is feasible to a high-throughput phenotype of the physiological information of crop leaves. However, there is no rapid quantitative and visual detection of free proline content in rice leaves under heavy metal stress.

The plant phenotyping based on hyperspectral imaging is a benefit for selecting the appropriate and a fast way to improve rice quality (such as yield) and to control heavy metal pollution. This study aimed to develop a rapid and accurate approach for high-throughput phenotyping of FP in rice leaves under Cd stress through hyperspectral imaging and chemometrics. Four Cd stress time points and five Cd concentration stress rice leaves were cultivated and tested. The rapid prediction models were established with full spectra and the screened effective wavelengths, respectively. The optimal quantitative detection model of FP was chosen to achieve visual distribution map of FP content.

## 2. Materials and Methods

### 2.1. Rice Leaf Samples

**Figure 1:**
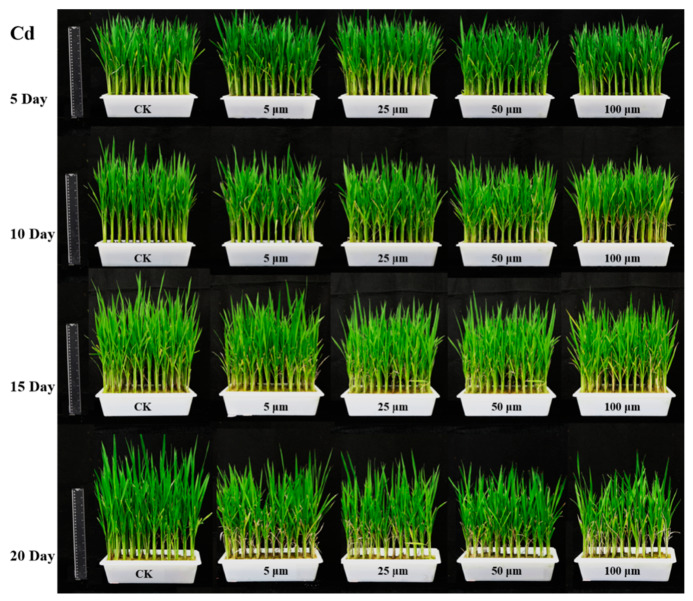
Rice plant growth under cadmium stress.

Rice leaf samples were cultivated by hydroponic culture. Rice seeds (*Xiushui* 134) were provided by Zhejiang Academy of Agricultural Sciences (Hangzhou, Zhejiang, China) and belong to single-season conventional late rice. A hydroponic experiment was carried out on Zijingang Campus (Zhejiang University, Hangzhou, China) in the summer of 2019. In order to reduce the incidence rate of rice plant growth, rice seeds were sterilized for 5 min and then disinfected with 1% sodium hypochlorite for 10 min. After washing for 5–6 times by sterile water, the rice seeds were placed in water for 2 days and were germinated in a 37 °C incubator. Nutrient solution replaced water after the germination. Rice seedlings with similar size plants (leaf length of approximately 6 cm) were selected to grow in total nutrient solution for one week and then treated with heavy metal cadmium stress at a certain concentration gradient. The incubator was adjusted to maintain keep a 14 h photoperiod with the temperature of 25 ± 1 °C and a 10 h night environment with temperatures of 20 ± 1 °C. Relative humidity was kept at 75%. CdCl_2_ was used to prepare rice cadmium stress solution at Cd^2+^ content with 0 μM (control group, CK), 5 μM, 25 μM, 50 μM and 100 μM. After 5d treatment, 80 rice plants in each group were selected randomly to provide the second leaves for hyperspectral imaging scanning. After hyperspectral information was obtained, the rice leaves were quickly cut, uniformly mixed, weighed and stored in a refrigerator at −80 °C to facilitate subsequent measurement of chemical reference values. For rice plants after 10 d, 15 d and 20 d of Cd stress treatment, the same procedure was performed. The rice plants are shown in Figure 1.

### 2.2. Hyperspectral Image Acquisition and Correction

Hyperspectral image data were acquired with the hyperspectral image spectrometer system, which could obtain visible and short wave near infrared spectroscopy from 400 to 1000 nm with spectral resolution 2.8 nm. The hyperspectral image spectrometer system consists of an imaging spectrometer (ImSpector V10E; Spectral Imaging Ltd., Oulu, Finland), a high performance CCD camera (Hamamatsu, Hamamatsu City, Japan) with a lens (OLES22; Specim, Spectral Imaging Ltd., Oulu, Finland), light source obtaining two 150 W tungsten halogen lamps (Fiber-Lite DC950 Illuminator; Dolan Jenner Industries Inc., Boxborough, MA, USA), an electronically controlled displacement platform (Isuzu Optics Corp., ChuPei, Hsinchu 30288, Taiwan), calibration whiteboard whose material is polytetrafluoroethylene, an black box providing interference-free environment, an hyperspectral image acquisition software (ITT, Visual Information Solutions, Boulder, CO, USA) and an ancillary computer [15]. The leaf samples were placed on a conveyor belt of the electronically controlled displacement platforms with a moving speed of 1.5 mm/s. The distance from the leaf sample to the CCD lens was 27.0 cm. The exposure time of the CCD camera was 0.14 s. These instrument parameters were adjusted to obtain clear and undistorted hyperspectral image. The whiteboard is primarily used for providing near 100% reflectivity. The lens was blocked to get an image with 0% reflectivity. This reflectance information was used to correct the light intensity.

Under the same Cd stress time in each group, hyperspectral images of 4 leaves were randomly collected, and the average spectra value of 4 leaves was taken as a leaf sample. A total of 100 samples were collected at 5 Cd stress concentrations and 4 stress times. The image size was 672 × 512 pixels. Due to the obvious noise in the front and back of the spectrum, 350 variables in the 500–950 nm region were selected as effective bands for rice leaf spectral analysis under heavy metal stress. Therefore, each hyperspectral image data is a three-dimensional 350 × 672 × 512 data block.

### 2.3. Gold Standard Methods for Measuring Leaf Contents of FP

Free proline content was detected according to the method of Bates et al. [16]. Of fresh rice leaves 0.5 g were placed in a 2 mL centrifuge tube containing 4 beads. The tube was immersed in liquid nitrogen and placed on a grinder for 1.5 min at 65 Hz. Of 3% sulfosalicylic acid solution 1500 μL was added to the centrifuge tube, and the leaves and sulfosalicylic acid were uniformly mixed by grinding on a grinder at 40 Hz for 30 s and put into a centrifuge at 10,000 rpm for 10min. The supernatant was extracted as the test liquid. The test liquid was subjected to a boiling water bath for 10 min, and after cooling, 750 μL of the supernatant was taken. Then 1000 μL of 10% acetic acid and 1500 μL of a 2.5% acid ninhydrin color developing solution were added into the supernatant. The mixture was boiled in a water bath for 40 min, cooled and 2500 μL of toluene was added and thoroughly shaken and mixed. The reaction solution was placed at a wavelength of 520 nm to measure the absorbance. The obtained absorbance values were combined with the relevant calculation formulas to calculate the corresponding samples of FP.

### 2.4. Data Analysis

Exploring the relationship between the spectral data and the FP content is the key for hyperspectral image analysis. Partial least squares (PLS), least-squares support vector machine (LS-SVM) and extreme learning machine (ELM) were used to explore the relationship between spectra data and FP content. Genetic algorithm (GA), partial least squares weighting regression coefficient (Bw) and competitive adaptive weighted sampling (CARS) were compared to select the variables highly related to FP content in rice leaves. The goal is to determine the FP content using the acquired spectra and the established calibration models. Models with better performances are preferable for real-world application in rice growth safety monitoring.

#### 2.4.1. Variable Screening Methods

GA is an intelligent computing model that simulates natural selection in Darwin’s theory of biological evolution. It has a strong global search capability, and can mine global optimal solutions from complex spectral peak information [17].

Bw is a set of regression coefficients that represent the weight of each spectral variable’s contribution to the result of full interactive verification [18]. The larger the absolute value of Bw, the more the corresponding wavelength is to facilitate the rapid quantitative detection of the target information content in the spectrum. Therefore, the spectral characteristic variables can be screened according to the PLS weighted regression coefficient. Usually draw the spectrum of the wavelength position of the spectrum and the Bw coefficient, and use the peak or valley as the characteristic wavelength.

CARS is also a feature variable selection method based on the PLS model weighted regression coefficients. Different from the method of screening feature variables by Bw, the CARS algorithm process is [19,20]: using Monte Carlo sampling and performing full interactive root mean square verification to establish a PLS model; retaining the larger value of the PLS weighted regression coefficient variables, remove the variables corresponding to the smaller value and finally use the remaining variables as the new set; repeating the above steps for the new set, after multiple calculations, select the wavelength corresponding to the set with the smallest root mean square error of cross validation (RMSECV) as the characteristic variable selected by CARS.

#### 2.4.2. Quantitative Analysis Methods

PLS is the most widely used machine learning method to explore the linear regression between the spectral data and the measured physiological and biochemical traits [21,22]. The important parameter in the PLS algorithm is the latent variable (LV) [23]. Choosing the appropriate LV is conducive to obtaining better model results. LVs are new variables formed by linear conversion of the original spectrum. The correlations between the new variables are orthogonal and uncorrelated, reducing various correlation interference between the original spectral variables, and some of the hidden variables can explain most of the original variable information. In the PLS model, the minimum RMSECV corresponds to the best LV [24].

LS-SVM is a machine learning method dealing with linear and non-linear data efficiently [25]. LS-SVM uses a hyperplane to fit the spectral data. The calculation process uses sparse approximation, robust regression and Bayesian inference [26]. Compared with the support vector machine [27], the solution difficulty is sampled, and the solution speed is improved, making it possible for LS-SVM to be suitable for online learning of large-scale and high-dimensional data.

ELM is a single hidden layer feedforward neural network, which is faster than the traditional neural network [28]. The ELM operation only needs to determine the threshold of the input layer and the hidden layer only one time, and does not need to be constantly readjusted [29]. In this study, the number of hidden layer nodes was set to the number of samples *n* in the training set. The model performs ELM calculation in order from 1 to *n*, and the minimum root mean square error of the prediction set is taken as the optimal ELM model and the number of hidden layer nodes.

### 2.5. Software and Model Evaluation

Hyperspectral images were firstly resized to reduce the data dimension by ENVI 4.6 (ITT, Visual Information Solutions, Boulder, CO, USA). The spectral data extraction and preprocessing, multivariate data analysis PLSR and image visualization were conducted on Matlab R 2014b (The Math Works, Natick, MA, USA). PLSR was performed on Unscrambler^®^ 10.1 (CAMO AS, Oslo, Norway).

The performances of the calibration models were evaluated by the coefficient of determination of calibration (*R_c_*), the coefficient of determination of prediction (*R_p_*), root mean square error of calibration and prediction (RMSECV and RMSEP) [30]. A better calibration model should have larger R, and lower root mean square error.

## 3. Results

### 3.1. FP Content of Rice Leaves

Table 1 shows the results of the mean values, standard deviation, maximum and minimum for FP content of the rice leaf samples in the five Cd concentration gradient treatment groups on different days. 

**Figure 2:**
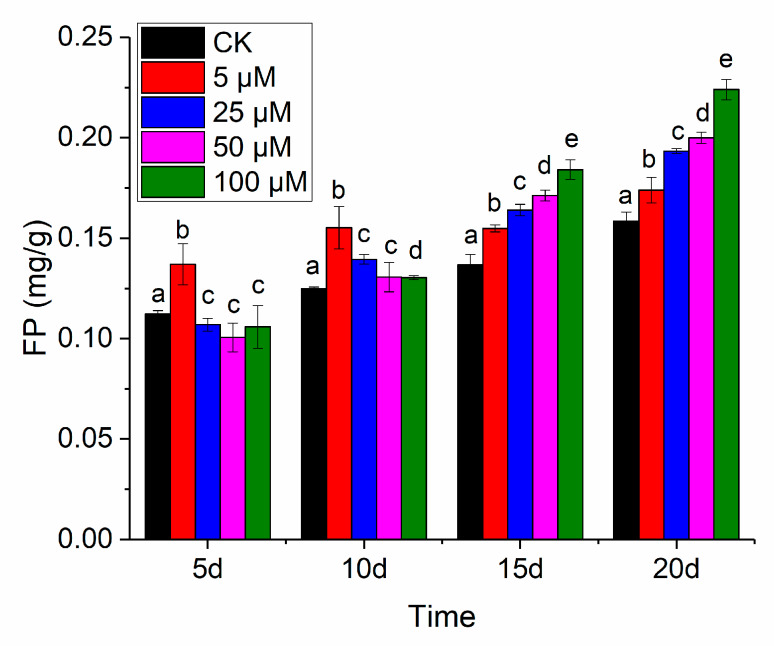
Changes in FP content under Cd stress, letters represent significant difference of *p* < 0.05.

The change trend of FP content with the difference of Cd stress time and concentration is shown in Figure 2. Different letters in the same day represents different significant differences of the *p* < 0.05 level in Figure 2, and the same letter in same day means there is no significant difference between the two Cd stress groups. In the early stage of heavy metal Cd stress (5d), compared with CK, the content of FP in mild stress (5 μM) increased, and the content of FP in other higher concentration stress groups decreased. With the increase of stress days (10 d), the content of FP in light stress (5 μM) was still the largest, and the content of FP in other groups also gradually increased, but the increase trend was slower, and it was not much different from CK. This may be because there was a certain balance between the FP and the corresponding oxide content in the plant. In the mid-stress period (15 d), the FP content was highest in severe stress (100 μM), and the FP content in the 25 μM and 50 μM stress groups continued to increase. This indicated that the leaves produced more FP to resist the stress of heavy metals, and there was a significant difference of the *p* < 0.05 level among the five groups with the different letters a, b, c, d and e. In the later period of stress (20 d), the FP content of the CK group was the lowest, and the FP content of other groups gradually increased with the increase of heavy metal stress concentration. This phenomenon was consistent with the relevant research pointed out that under stress environment (such as drought, high temperature, heavy metals, etc.), the content of proline would increase [31,32]. In terms of significant differences, as a whole, with the gradual increase of stress time, the differences between different heavy metal stress groups gradually changed from insignificant differences to significant differences.

### 3.2. Spectra Analysis

**Figure 3:**
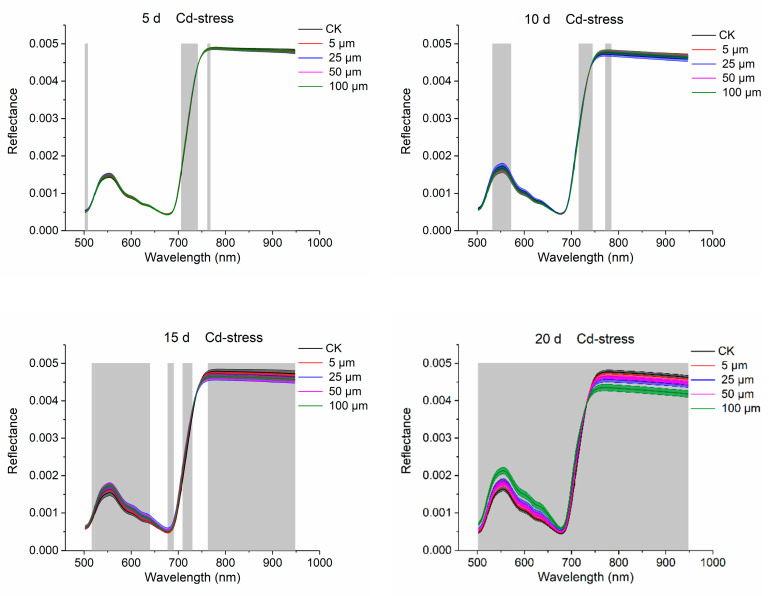
Variation trend on **5 d**, **10 d**, **15 d** and **20 d** of rice leaves under Cd stress.

The average spectrum with standard deviation of rice leaves under the stresses of different cadmium are presented in Figure 3. Typical spectral curves of green leaves between 500 and 955 nm could be observed. As the time of Cd stress increased, differences among spectral curves of samples under the Cd stress of different concentrations became larger. The differences became obvious when the stress time came to 15 days and 20 days. The ANOVA was also calculated between the independent wavelength variables and the stress degrees. Wavelengths with *p* < 0.05 are considered as important wavelengths relating to the stress degrees. With the increase of stressing time, differences of more wavelength variables among different treatments became significant. When the stressing time came to 20 d, significant differences could be found in all wavelengths. Seen from Figure 3, the wavelengths lying in the range of 690–720 nm showed significant differences an all four images, illustrating an earlier or more consistent indication of stress [33]. These wavelengths have been reported as indicators of plant stresses. The wavelengths that had a significant difference on 5 d without significant on 10 d existed. This may be because as the stress time increases from 5 d to 10 d, the differences of some substances characterized by this band are masked by the information of other substances.

### 3.3. Quantitative Analysis Based on Full Spectra

At present, there are few studies on the rapid detection of FP content in rice leaves, which may be related to the characteristics of low FP content and poor stability. The full spectra contain a wealth of plant leaf information, which can fully reflect the optical characteristics of the leaves. The relationship between FP content and the full spectra from the hyperspectral image data was explored by building PLS, LS-SVM and ELM models. To establish the regression models, the samples were split into the calibration set and the prediction set at the ratio of 3:1 (75 samples for calibration and 25 samples for prediction). The samples were ranked from low to high according to their physiological parameters, and the third sample of every four samples was selected into the prediction set, and the remaining three samples of every four samples were selected into the calibration set. For both PLS, LS-SVM and ELM, leave-one-out cross validation were conducted to optimize the model performances. 

Table 2 shows the prediction results of FP concentrations by PLS, LS-SVM and ELM models. When building a mathematical model, better prediction performance is the first choice. ELM has the best prediction performance, with *R_p_* reaching 0.9101 and RMSEP of 0.0161 mg/g, the optimal number of hidden layer nodes was 38.

### 3.4. Quantitative Analysis Based on Selected Variables

The spectrum obtained by the hyperspectral image acquisition system has the characteristics of a wide frequency band, and the groups in the physiological indexes of rice leaf stress are selective for the absorption of light energy. How to select some effective variables related to the physiological index of rice leaf stress from the total variable information and eliminate irrelevant redundant variables is of great significance for simplifying the calculation process and establishing a model with high prediction accuracy and robustness in the later stage. Therefore, this study used GA, CARS and Bw to screen the characteristics of hyperspectral information that were highly related to the physiological index of rice leaf stress, and built the rapid quantitative detection models based on the selected characteristic wavelength.

The results of the quantitative detection model established after variable screening of rice leaf FP under Cd enhancement are shown in Table 3. GA, CARS and Bw selected 29, 27 and 14 variables for the establishment of PLS, LS-SVM and ELM models, respectively. The best model based on variables selected by GA, CARS and Bw is all ELM models with *R_p_* 0.8929, 0.9426 and 0.8995, respectively. The ELM model based on 27 variables selected by CARS is a big improvement over the full-spectrum prediction accuracy and better prediction performance than the 29 variables screened by GA and 14 variables screened by Bw. This shows that the 27 variables selected by CARS are the optimal characteristic wavelengths, further removing the invalid information and noise in the full spectra, retaining the effective information to the greatest extent, and showing a stronger ability to predict the FP content of rice leaves than the 350 variables in the full spectra. Compared with the full spectra best model (*R_p_* is 0.9190), the model prediction effect is enhanced, which shows the CARS screening method remove most of the invalid information and noise in the full spectra, and retain valid information.

From the perspective of detection accuracy, the rapid detection model of the optimal FP content of rice leaves under Cd stress was the ELM model (*R_p_* was 0.9426) established based on 27 variables selected by CARS. The 27 variables screened by CARS were 528.07, 541.67, 568.96, 625.18, 637.75, 650.34, 662.97, 665.5, 674.35, 688.29, 746.95, 762.35, 763.63, 784.23, 785.52, 795.84, 797.13, 812.65, 832.11, 833.41, 869.9, 871.21, 897.41, 898.72, 910.55, 928.99 and 943.52 nm. From the perspective of simplified detection, the optimal fast detection model was an ELM model (*R_p_* was 0.8995) established on 14 variables selected by Bw. The 14 variables selected by Bw were 502.21, 539.19, 601.37, 626.44, 650.34, 670.55, 689.56, 709.91, 729.04, 764.92, 836.01, 900.04, 915.81 and 943.52 nm. Overall, these three variable screening methods greatly simplified the input variables of the model. The 29 variables screened by GA were 593.88, 595.13, 598.87, 596.38, 600.12, 626.44, 621.42, 597.62, 625.18, 623.93, 627.69, 622.67, 592.63, 620.42, 601.37, 628.95, 591.38, 602.63, 905.29, 504.66, 505.89, 618.91, 630.2, 690.83, 711.18, 521.14, 545.38, 872.52 and 906.6 nm.

### 3.5. Visual Analysis of FP

**Figure 4:**
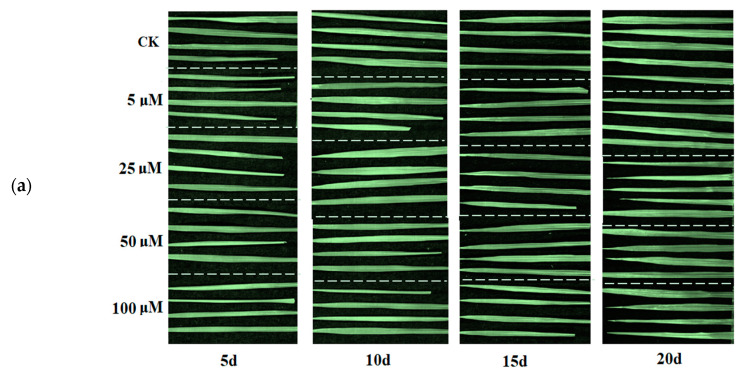

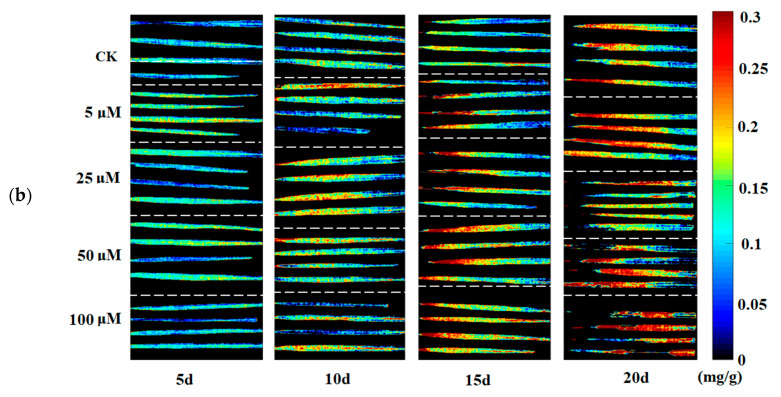
RGB image (**a**) and FP content visualization map (**b**) of rice leaves under Cd stress.

The hyperspectral image acquired the spectral information of each pixel. This feature is conducive to the prediction of the physiological index content of each pixel, thereby forming a visual distribution map of the physiological index content on the surface of the rice leaf. First, the same preprocessing and variable screening on the spectrum of each pixel of the hyperspectral image were performed same with the spectrum of the rapid detection model in this paper. Then the spectrum of each pixel was introduced into the FP rapid detection model obtained in 3.4 to obtain each pixel point predicted FP content. To simplify the computing process, the ELM model of 27 variables screened by CARS for FP prediction under Cd stress was used. Finally, the map of FP content predicted by the spectrum of each pixel was processed with pseudo-color and 5 d, 10 d, 15 d and 20 d stress cycle and five Cd stress concentrations of the FP content of the rice leaf visualization map was obtained in Figure 4.

It can be clearly seen from the visualization in Figure 4 that FP content varied with the concentration of heavy metal Cd and the number of stress days. The FP content of rice leaves under 5 μM Cd stress was the highest at 5 d, which was related to the result in Figure 2 and another study [34] that showed that trace heavy metals could stimulate plant growth. With the prolongation of the time of Cd stress, the FP content of rice leaf tips under Cd stress increased with the increase of Cd stress concentration. At 20 d, there was also a large amount of FP in the petioles of the most severe Cd stress group (100 μM). On the whole, the visual distribution map based on hyperspectral imaging technology could intuitively provide spatiotemporal information on the difference between FP, an important stress physiological index between rice leaf and leaf, which helps to monitor the growth status of plants.

## 4. Discussion

### 4.1. Influence of Cd Stresses on Rice Leaves

FP content analysis in Table 1, Figure 2 and Figure 4 indicated that with the extension of time, the variations of rice leaves under stresses of different concentrations of Cd became larger. Figure 2 and Figure 4 shows that FP content increased with increasing Cd stress time. This phenomenon illustrates that as the heavy metal Cd aggravating the growth of rice, some antioxidant active ingredients such as FP would be generated in the leaf tissues to resist the damage of heavy metal Cd. Exposure to Cd results in the generation of reactive oxygen species such as O_2_^−^, ^.^OH and ^1^O_2_ in plant tissues [35]. FP is an important substance used in osmotic regulation in plant cells to protect the cell membrane system, maintain the structure of intracellular enzymes, and reduce the degradation of intracellular proteins [36]. Under normal circumstances, FP content in plants is relatively low, which is similar with in Table 1, Figure 2 and Figure 4 (CK). Under stressful conditions (such as drought, high temperature, heavy metals, etc.), FP will increase significantly. Through Figure 4, the changes and differences of antioxidants in leaves under Cd stress can be quickly and visually determined. Average reflectance spectra with standard deviation of rice leaves in Figure 3 visually show the effect of Cd stress time on the optical properties of the leaves. On the 20th day, the spectra of rice leaves with different stress concentrations showed significant differences in the whole band. 

### 4.2. Advantages of Hyperspectral Imaging

Non-imaging reflectance spectra have been utilized for plant phenotyping in previous studies [37,38,39]. Recent studies focused more on applying imaging reflectance spectra for plant phenotyping [40,41,42]. Non-imaging reflectance spectra are generally obtained from the point scan, repeated measurements from different regions were conducted to obtain reflectance spectra, which could represent the sample. Compared with non-imaging reflectance spectra, hyperspectral imaging can acquire spectral features within the entire sample not one point of the sample, thus hyperspectral images contained more information than non-imaging. In this study, prediction maps are a merit from the imaging feature of hyperspectral imaging. The established models were applied to each pixel within the hyperspectral image. Prediction maps with predicted features presented by color gradients can be used to explore the distribution of specific features.

Figure 4 vividly shows the distribution of FP content in leaves. Regarding the normal growth of rice leaves, the CK groups in four time periods showed the normal growth of rice without heavy metal stress, and the distribution diagram showed a slight increase trend with the increase of growth time. The content of FP in rice leaves was basically kept below 0.15 mg/g before 15 d, and the content of FP in some leaves exceeded 2 mg/g in 20 d. As for the impact of Cd stress time, as the time increased, the FP content of each group showed increasing trends. The heavy metal stress was the most serious, and the FP concentration reached the maximum in 20 d. For the effect of Cd stress concentration, the FP content in the 5 μM group was largest in 5 d [43]. The FP content of the 10 μM, 15 μM and 20 μM groups began to increase in 10 d, indicating that rice leaves began to produce more FP to resist heavy metal damage. Both 15 d and 20 d showed the greater concentration of Cd, the higher FP content in rice leaves. For the difference in leaf surface distribution, FP content in the leaf tip was higher than that of the petiole, indicating that the FP resistance mechanism of the leaf tip was more sensitive to the damage of heavy metals. Overall, hyperspectral imaging helps to extend ‘knowing the regional value’ to ‘knowing the pixel-wise distribution’. Hyperspectral imaging offers the possibility for rapid and accurate exploration and visualization of phenotypes at the pixel-wise level, leaf level and plant level.

### 4.3. Prediction Models

The prediction of FP content by reflectance spectra were merely studied. Traditionally, ultraviolet-visible spectrophotometers or the automatic microplate reader [44,45] were used to measure FP content in plants. These methods were destructive, complex to operate and had low efficiency. Besides, they needed quite complicated sample preparation and various reagents. The rapid, cost-effective and nondestructive hyperspectral imaging had significant potential to overcome these shortcomings. With no or minimum sample preparation, acquisition and analysis of hyperspectral images can be designed as automation, saving labor and extending large scale application. The key factor was the prediction models.

In this study, three models, PLS, LS-SVM and ELM were used to build prediction models. PLS were efficient in dealing with linear issues while LS-SVM can treat both linear and non-linear issue well. For real-world application, models with better performances were preferred. With better performances, the prediction would be more accurate and reliable. Both the extracted reflectance spectra and the measured reference physiological parameter FP could affect the results. In all models, ELM detection performance is better than PLS and LS-SVM models. It shows that the ELM model not only has fast training speed and good generalization ability, but also has certain advantages in detecting the relationship between FP content in rice leaves and Cd stress and hyperspectral information.

To improve model robustness and performances, wavelengths selection has been widely applied in spectral data analysis of near-infrared spectroscopy and hyperspectral imaging. We used GA, CARS and Bw methods to explore the relative importance of wavelengths contributing to the prediction of FP. The optimal feature variables are the 27 variables selected by CARS with best *R_p_*. Both CARS and Bw screened 650.34 and 943.52 nm; CARS and GA selected 625.18 nm and Bw and GA chosen 601.37 and 626.44 nm. These same wavelengths have a stronger correlation with the FP content in rice leaves, and need to be further analyzed and applied. Free proline is a cyclic imino acid composed of a pyrrolidine and a hydroxy acid. The selected wavelength 601.37, 625.18, 626.44, 650.34 or 943.52 nm may be related to the composition of FP, for example, it may be related to the doubling or fundamental frequency of -OH in hydroxy acid.

In spectral data analysis of near-infrared spectroscopy and hyperspectral imaging, there were various ways to improve the model performances. Outlier samples removal was an effective approach [46]. Both the extracted reflectance spectra and the measured reference physiological parameters could affect the results. In this study, we analyzed the data by ‘trusting’ that there were no outlier samples. In the future studies, this approach can be tried to improve the model performances. At present, studies of using reflectance spectra for physiological parameter FP prediction were limited to few research. Methods to improve the model accuracy and robustness needed to be further studied. Taking the mostly studied physiological parameter chlorophyll as an example, various methods such as full spectra, specific wavelengths and spectral indices have been developed to estimate chlorophyll concentrations for better meeting the application of farmland scenes [47]. As a consequence, the ultimate goal was to use hyperspectral imaging combining with the established models as standard methods to estimate FP concentration as well as other physiological parameter in real-world application.

## 5. Conclusions

In conclusion, hyperspectral imaging was successfully utilized for high throughput phenotyping of FP in rice leaves under Cd stress. Changes in spectra with stress time and concentration showed the optical properties of rice leaves. Good performances of prediction models using two modeling approaches (models using full spectra and models using selected wavelengths) with *R_p_* 0.9190 and 0.9426 for FP content demonstrated the advantage of hyperspectral imaging with machine learning algorithm for simultaneous estimation of multi-physiological parameters in a rapid, cost effective and non-destructive manner. The optimal quantitative detection model is ELM model based on 27 variables selected by CARS with *R_p_* 0.9426 was used to obtain a visual distribution map of FP content in rice leaves. The distribution map of FP provides an efficient way to simultaneously visualize the multi-physiological parameters within rice leaves. The study ultimately provided technical means for real-time and high-throughput screening of FP content in rice leaf under heavy metal stress to precise manage of rice growth security accurately.

## Figures and Tables

**Table 1:** Results of chemical values of free proline (FP) in rice leaves under cadmium (Cd) stress.

Indicators	Groups	5 d	10 d	15 d	20 d
FP	Number	25	25	25	25
	Min	0.0740	0.1170	0.1304	0.1401
	Max	0.1359	0.1479	0.1795	0.2186
	Mean	0.1027	0.1335	0.1622	0.1880
	S.D.	0.0172	0.0096	0.0138	0.0192

**Table 2:** Results of rapid detection models for FP content in rice leaves under Cd stress based on the full spectra.

Models	Parameter ^1^	*R_c_*	RMSECV (mg/g)	*R_p_*	RMSEP (mg/g)
PLS	9	0.8915	0.0015	0.8830	0.0191
LS-SVM	246,566.22; 30,674.42	0.9292	0.0123	0.8541	0.0215
ELM	38	0.9435	0.0109	0.9190	0.0161

^1^ The parameters of PLS model are the optimal LV, the parameters of LS-SVM model are gam and sig2, and the parameters of ELM model are the optimal number of hidden layer nodes.

**Table 3:** Models for detection of FP under Cd stress based on the characteristic wavelength.

Ways	Number	Models	Parameter ^1^	*R_c_*	RMSECV (mg/g)	*R_p_*	RMSEP (mg/g)
GA	29	PLS	11	0.8686	0.0164	0.8725	0.0199
		LS-SVM	1,168,705.8; 6596.5	0.9131	0.0135	0.8498	0.0214
		ELM	28	0.9388	0.0114	0.9219	0.0166
CARS	27	PLS	9	0.8850	0.0154	0.8905	0.1840
		LS-SVM	661,182.0; 1889.8	0.9356	0.0117	0.8590	0.0214
		ELM	24	0.9401	0.0112	0.9426	0.0135
Bw	14	PLS	7	0.8959	0.0147	0.8765	0.0196
		LS-SVM	2,430,985.3; 4068.6	0.9370	0.0116	0.8574	0.0213
		ELM	19	0.9352	0.0117	0.8995	0.0178

^1^ The parameters of the PLS model are the optimal LV, the parameters of the LS-SVM model are gam and sig2, and the parameters of the ELM model are the optimal number of hidden layer nodes.
